# Biomarkers of Response to Biologic Therapy in Juvenile Idiopathic Arthritis

**DOI:** 10.3389/fphar.2020.635823

**Published:** 2021-02-02

**Authors:** Varvara Choida, Margaret Hall-Craggs, Bethany R. Jebson, Corinne Fisher, Maria Leandro, Lucy R. Wedderburn, Coziana Ciurtin

**Affiliations:** ^1^ Centre for Adolescent Rheumatology Versus Arthritis at UCL UCLH and GOSH, Division of Medicine, University College London, London, United Kingdom; ^2^ Department of Adolescent Rheumatology, University College London Hospital, London, United Kingdom; ^3^ Centre for Medical Imaging, University College London, London, United Kingdom; ^4^ University College London Great Ormond Street Institute for Child Health, London, United Kingdom; ^5^ NIHR Biomedical Research Centre at Great Ormond Street Hospital, London, United Kingdom

**Keywords:** juvenile idiopathic arthritis, serological biomarkers, imaging biomarkers, cellular biomarkers, biologics

## Abstract

**Background:** Juvenile idiopathic arthritis (JIA) is the most common chronic inflammatory arthritis of childhood, characterized by various clinical phenotypes associated with variable prognosis. Significant progress has been achieved with the use of biologic treatments, which specifically block pro-inflammatory molecules involved in the disease pathogenesis. The most commonly used biologics in JIA are monoclonal antibodies and recombinant proteins targeting interleukins 1 (IL-1) and 6 (IL-6), and tumor necrosis factor α (TNF-α). Several biomarkers have been investigated in JIA.

**Aims:** To assess the level of evidence available regarding the role of biomarkers in JIA related to guiding clinical and therapeutic decisions, providing disease prognostic information, facilitating disease activity monitoring and assessing biologic treatment response in JIA, as well as propose new strategies for biologic therapy-related biomarker use in JIA.

**Methods:** We searched PubMed for relevant literature using predefined key words corresponding to several categories of biomarkers to assess their role in predicting and assessing biologic treatment response and clinical remission in JIA.

**Results:** We reviewed serological, cellular, genetic, transcriptomic and imaging biomarkers, to identify candidates that are both well-established and widely used, as well as newly investigated in JIA on biologic therapy. We evaluated their role in management of JIA as well as identified the unmet needs for new biomarker discovery and better clinical applications.

**Conclusion:** Although there are no ideal biomarkers in JIA, we identified serological biomarkers with potential clinical utility. We propose strategies of combining biomarkers of response to biologics in JIA, as well as routine implementation of clinically acceptable imaging biomarkers for improved disease assessment performance.

## Introduction

Juvenile idiopathic arthritis (JIA) is a heterogeneous group of diseases, with onset before the age of 16. JIA has been divided into seven subtypes with distinct clinical presentations, according to the International League of Associations for Rheumatology (ILAR) classification criteria (Petty et al., 1998). More specifically, the categories are systemic-onset JIA (SJIA), persistent or extended oligoarticular JIA, polyarticular rheumatoid factor (RF) positive and polyarticular RF negative JIA, enthesitis-related arthritis (ERA), psoriatic arthritis (PsA) and undifferentiated arthritis. There is a variety of composite scores and outcomes to quantify and monitor the disease activity in JIA (McErlane et al., 2013). The Juvenile Arthritis Disease Activity Score (JADAS) is a composite score consisted of the physician and patient/guardian global assessment (visual analogue scale 0–10 cm), the number of active joints and the normalized values of C-reactive protein (CRP) or erythrocyte sediment ratio (ESR) out of 10 (Consolaro et al., 2016). The American College of Rheumatology (ACR) Pediatric response criteria (ACR Pedi 30/50/70 and 90) evaluate 30, 50, 70, and 90% improvement in response to treatment, respectively (Giannini et al., 1997; Ruperto et al., 1998). They include two additional core outcome variables to JADAS: the number of limited joints and functional ability, measured by the Childhood Health Assessment Questionnaire (CHAQ). There are established definitions for inactive disease, clinical remission on treatment (inactive disease for ≥6 months) and off treatment (inactive disease for ≥12 months) (Wallace et al., 2004), as well as for flares (Brunner et al., 2002). However, these definitions do not work equally well for all JIA subtypes because of the heterogeneity of patients’ clinical presentation, and alternative definitions have surfaced and used as outcomes in research studies (Heiligenhaus et al., 2012; Consolaro et al., 2016; Zanwar et al., 2018).

The emergence of biologic treatments has changed the prognosis for many JIA patients, whose condition did not improve adequately on conventional synthetic disease modifying anti-rheumatic drugs (csDMARDs), mainly methotrexate, or experienced side effects because of them. TNF-α inhibitors, such as etanercept (human dimeric fusion protein which functions as a decoy receptor and binds to soluble TNF-α), adalimumab (human monoclonal antibody -mAb- which binds with high affinity both soluble and membrane-bound TNF-α) and infliximab (chimeric mAb which blocks both soluble and trans-membrane TNF-α) are widely used in JIA. In fact, etanercept is one of the most frequently prescribed biologics for JIA in many countries, including the United Kingdom (Geikowski et al., 2014; Kearsley-Fleet et al., 2020). Other biologics include tocilizumab (humanized mAb which blocks both soluble and trans-membrane IL-6), anakinra (human IL-1 receptor agonist which blocks IL-1 type 1 receptor) and canakinumab (human mAb against IL-1β), abatacept (human cytotoxic T-lymphocyte-associated protein 4 immunoglobulin fusion protein, acting as T-cell co-stimulatory blockade) and rituximab, a chimeric anti-CD20 mAb causing B-cell depletion. The efficacy of biologics varies depending on the disease subtype, although there is lack of head-to-head clinical trials between different biologics (Davies et al., 2017). Despite the positive short-term outcomes in numerous studies (Ungar et al., 2013), many patients switch biologics due to primary inefficacy, loss of response or adverse effects. Data from biologic registries in the United Kingdom, suggest that 23% of patients receive at least two biologic drugs, 5% at least three and 1% four or more biologic drugs within a median observational period of 2.2 years (Kearsley-Fleet et al., 2020). The retention rate of biologics declines with time, from 92.9% in the first year of treatment to 68.1% at 4 years, according to a Portuguese registry (Mourao et al., 2016). About one third of JIA patients retained their first anti-TNF treatment in 10 years, according to a local registry (Favalli et al., 2017). In addition, tapering or discontinuation of biologic treatment is a reasonable option in the context of clinical remission. Unfortunately, in many cases treatment requires to be resumed, due to worsening disease control. Therefore, there is a great need for biomarkers to guide clinical decisions, such as commencing, switching or tapering biologic DMARDs (bDMARDs).

Biomarkers are characteristics that are objectively measured and indicate the presence or severity of a disease state. Therapeutic biomarkers reflect biological, pathogenic or pharmacologic processes as indicators of a therapeutic effect, whilst surrogate markers are biomarkers that serve as a substitute for a clinically meaningful endpoint and can provide evidence to help predict the effect of a therapeutic intervention (Hunter et al., 2010). In this review, we present the results of a comprehensive search of the literature via PubMed in order to identify clinical, serological, genetic, cellular and imaging biomarkers which can assist clinicians in their efforts to personalize bDMARDs prescription and adjust treatment strategies for JIA patients in a judicious manner. As the largest body of evidence regarding potential biomarker utility is related to treatment with etanercept in JIA, and SJIA represents the most severe JIA phenotype, we will be dedicating particular attention to studies investigating this specific treatment and disease type.

## Baseline Clinical Characteristics of JIA as Predictors of Response to Biologic Treatments

There have been multiple studies, comprising large number of patients, which assessed the baseline characteristics as predictors of response to etanercept, which has been one of the best studied biologic treatments in JIA (Table 1). Various patient characteristics, such as lower CHAQ scores reflecting better functional levels (Otten et al., 2011a; Geikowski et al., 2014; Kearsley-Fleet et al., 2016), lack of concurrent steroid treatment (Geikowski et al., 2014; Kearsley-Fleet et al., 2016) and younger age (Solari et al., 2013; Geikowski et al., 2014; Kearsley-Fleet et al., 2016) appeared to be favourable characteristics for successful treatment with etanercept. Patients with SJIA were less likely to have a positive response to etanercept, compared to other JIA types (Otten et al., 2011a; Geikowski et al., 2014), whereas the persistent oligoarticular type was associated with the highest response rate to etanercept (Alexeeva et al., 2017). Interestingly, shorter disease duration was a positive predictor of therapeutic benefit (Kearsley-Fleet et al., 2016; Mo et al., 2020) in contrast to the number of DMARDs used before the initiation of etanercept treatment, which was associated negatively with treatment response (Otten et al., 2011a). Taken together, these findings support the use of etanercept early in the disease course for non-systemic JIA.

**TABLE 1:** Baseline clinical, serological and therapeutic characteristics as predictors of response to etanercept in JIA.

Ref.	Study design	N patients	Results
Geikowski et al. (2014)	Prospective observational multi-centre	863	Baseline predictors of ACR Pedi 70 after 6 months of treatment were:
			•High ESR (OR 1.02; 95% CI 1.01, 1.03)
			•Lower CHAQ-DI (OR 0.70; 95% CI 0.56, 0.88)
			•Lower age at start of treatment (OR 0.94; 95% CI 0.91, 0.98)
			•Treatment without corticosteroids (OR 0.68; 95% CI 0.49, 0.94)
			•Any JIA type other than SJIA (OR 0.28; 95% CI 0.16, 0.52), model AUC 0.646
Kearsley-Fleet et al. (2016)	Prospective observational multi-centre	496	Baseline predictors of ACR Pedi 90 after 1 year of treatment were:
			•Shorter disease duration (OR 0.91; 95% CI 0.85, 0.97)
			•Lack of concurrent steroid treatment (OR 0.57; 95% CI 0.35, 0.93)
			•History of chronic anterior uveitis (OR 2.26; 95% CI 1.08, 4.71)
Otten et al. (2011a)	Prospective observational multi-centre	262	Baseline predictors of excellent response, compared to intermediate or poor response^*^ after 15 months of treatment were:
			•Lower CHAQ score (OR 0.49; 95% CI, 0.33–0.74),
			•Low number of DMARDs (including methotrexate) used before introduction of ETN (OR 0.64; 95% CI, 0.43–0.95),
			•Younger age of disease onset (OR 0.92; 95% CI, 0.84–0.99).
Mo et al. (2020)	Retrospective single-centre	87	A machine learning model to predict response (AUC 79.17%) included:
			•Tender joint count
			•Time interval (disease onset to treatment initiation),
			•Lymphocyte count
			•Weight
Solari et al. (2013)	Retrospective single-centre	173	Predictors of inactive disease were:
			•Age at disease onset<3.6 years [HR 1.61 (1.04–2.49)]
			•Absence of wrist involvement [HR 2.19 (1.38–3.48)]
Alexeeva et al. (2017)	Prospective open-label	197	Clinical phenotype predicted response:
			•More patients with persistent oligoarticular (65.5%) vs. RF negative polyarticular (23.4%) or ERA (38.5%) achieved an excellent response to treatment at 1 year.
Su et al. (2017)	Retrospective single-centre	58	CID at 6 months post-treatment was not predicted by:
			•Age of disease onset
			•Gender
			•JIA subtypes (only extended oligoarticular, polyarticular, SJIA included)
			•Number of active joints at disease onset
			•Duration from disease onset to starting treatment
			•ESR, CRP, and CHAQ scores.
			No difference in IL- 12p70, TNF-α, IL-10, IL-6, and IL-1β levels before and 6 months post ETN treatment, between the patients who achieved or not CID at 6 months

ACR, American college of rheumatology; AUC, area under the curve; CHAQ-DI, childhood health assessment questionnaire disability index; CI, confidence interval; CID, clinically inactive disease; CRP, c-reactive protein; DAS, disease activity score; DMARDs, disease modifying anti-rheumatic drugs; ESR, erythrocyte sediment ratio; ETN, etanercept; ERA, enthesitis-related arthritis; HR, hazard ratio; (95% confidence interval); IL, interleukin; ILAR, International League of Associations for Rheumatology; JADAS, juvenile arthritis disease activity score; JIA, juvenile idiopathic arthritis; OR, odds ratio; Pedi, pediatric; RF, rheumatoid factor; TNF, tumor necrosis factor.

Data from 62 polyarticular JIA patients who completed a long extension clinical trial of adalimumab, suggested that the achievement of JADAS-27 (assessing 27 joints) clinical remission was more likely in early responders, who met either the ACR Pedi 50 or above response criteria or JADAS-27 threshold for inactive or low disease activity at 4, 8, 12 and 16 weeks (Lovell et al., 2020). Patients with ERA who had raised body mass index (BMI) were less likely to achieve inactive disease after 1 year irrespective of treatments, including biological agents; 19/72 of ERA patients were on anti-TNF treatment (Makay et al., 2016).

## Therapeutic Drug Monitoring and Anti-drug Antibodies as Biomarkers of Efficacy and Toxicity of Biologic Treatments in JIA

The clinical utility of TDM and measurement of ADA has been investigated intensively in patients with inflammatory bowel disease (IBD), predominantly in relation to infliximab and adalimumab (Papamichael et al., 2019). Monitoring of trough concentrations and ADA can be 1) proactive, in order to titrate dosing, with a view to improving clinical outcomes and drug survival, or 2) reactive, to guide decisions upon the emergence of secondary loss of response (SLR). For example, a retrospective study in ulcerative colitis showed that patients who developed SLR on adalimumab or infliximab, despite adequate trough levels, had longer duration of response when switched to a different class of biologics compared to receiving a different anti-TNF-α agent (Yanai et al., 2015).

The formation of ADA is documented with all the licensed biologic treatments in JIA. However, the relation between ADA and treatment failure or adverse effects, the persistent or transient nature of ADA, as well as their prevalence in relation to treatment duration, vary across the different biologics (Doeleman et al., 2019). For instance, antibodies against etanercept, abatacept and canakinumab are non-neutralizing and are not linked with loss of efficacy (Mori et al., 2012; Sun et al., 2016; Doeleman et al., 2019; Verstegen et al., 2020). Similarly, despite the increased prevalence of ADA in patients treated with anakinra (82% at 12 months), the majority of patients develop non-neutralizing antibodies and do not lose treatment response (Lovell et al., 2000; Ilowite et al., 2009). In comparison, adalimumab and infliximab ADA are associated with reduced trough levels and loss of efficacy (Ruperto et al., 2010; Skrabl-Baumgartner et al., 2015; Marino et al., 2018; Brunelli et al., 2020). Although the prevalence of tocilizumab ADA is low in JIA, 43% of patients with neutralizing ADA experienced treatment failure compared with 6% of JIA patients with no detectable ADA (Brunner et al., 2015). Concomitant use of methotrexate has a protective role against the development of adalimumab ADA [risk ratio 0.33; 95% Confidence interval (95% CI) 0.21, 0.52] (Doeleman et al., 2019). The above findings regarding adalimumab were also reported in relation to patients receiving this drug for JIA-associated uveitis (Skrabl-Baumgartner et al., 2019). In addition, the risk of infusion reactions in patients treated with tocilizumab, infliximab or rilonacept increased in the presence of ADA (Ruperto et al., 2007; Lovell et al., 2013; Yokota et al., 2014).

In conclusion, there is a potential clinical role of monitoring ADA and trough concentrations, especially in patients receiving adalimumab and infliximab monotherapy (Doeleman et al., 2019). However, detecting biologic drug trough levels is not always practical, especially for patients who self-administer their medication subcutaneously as their blood tests should be coordinated prior to their next dose administration. Moreover, establishing concentration thresholds for therapeutic benefit is challenging, because results are likely to vary depending on the selected assays, clinical endpoints, or even type of JIA.

## Potential Role of Measuring Proinflammatory Proteins in Serum as Biomarkers of Therapeutic Response to Biologic Treatments in JIA

The myeloid-related S100 proteins (low molecule proteins named S100 as they are soluble in 100%, i.e., saturated, ammonium sulfate at neutral pH): S100A12 and the S100A8/S100A9 complex (also known as myeloid-related protein 8/14–MRP8/14 or calprotectin) are proinflammatory proteins secreted by myeloid cells. This family of proteins have been widely investigated in the rheumatological field and have shown significant utility as biomarkers to predict disease severity, response to treatment and disease flare in conditions including rheumatoid arthritis (RA), systemic lupus erythematosus (SLE) and JIA (Soyfoo et al., 2009; Nordal et al., 2016). Their efficacy as potential biomarkers for response to treatment in JIA was initially investigated in patients treated with methotrexate monotherapy. A prospective study of 87 patients, with all types of JIA represented, demonstrated that patients with higher MRP8/14 levels before initiating methotrexate were more likely to have a better response from treatment at 6-months follow-up (Moncrieffe et al., 2013). Similarly, a multi-centre study, including 88 patients from three national biologic registries who received etanercept or adalimumab as their first biologic treatment, showed that baseline MRP8/14 levels were significantly higher in responders compared to non-responders (Anink et al., 2015). Treatment response was defined as achieving at least ACRPedi50 response within 6 months of treatment. Levels above 1,193 ng/ml predicted treatment efficacy of anti-TNF biologic with 66% sensitivity, 81% specificity and an area under the curve (AUC) of 0.76. Furthermore, there was a greater reduction in the levels of MRP8/14 in patients who achieved inactive disease vs. those who did not. In the same patient cohort, S100A12 baseline levels were also higher in patients who met the treatment response criteria (Gohar et al., 2018). A concentration above 213 ng/ml predicted a minimum ACRPedi50 response with 58.6% sensitivity and 80.6% specificity (AUC = 0.734). Moreover, the mean S100A12 levels decreased significantly after 4 weeks of etanercept treatment in 21 patients with polyarticular and oligoarticular JIA (Foell et al., 2004). When tested alone or incorporated into multivariate models, S100A8/S100A9 proteins have shown higher predictive power when determining treatment response than clinical variables such as ESR or CRP (42, 44). However recently, a Dutch study involving 123 patients with early non-systemic JIA, mostly RF negative polyarticular subtype, reported no difference in baseline MRP8/14 levels between responders (patients who achieved at least a ACRPedi50 response) and non-responders, though patients in this study received different DMARDs (Barendregt et al., 2020). Another observational study which measured the baseline levels of MRP8/14 in 152 non-systemic JIA patients before starting anti-TNF treatment, demonstrated that patients who reached inactive disease at 12 months had higher levels compared to patients who did not (Alberdi-Saugstrup et al., 2017). However, a cut-off concentration of 500 ng/ml was associated with very low sensitivity of predicting inactive disease and discontinuation due to lack of efficacy (22 and 39% respectively), with specificities of 80 and 83% respectively. At the same time, the selected cut-off level could not predict treatment response based on any of the ACR Pedi criteria.

Long-lasting efficacy is a desirable outcome of biologic treatment in JIA. A potentially promising biomarker for long-term retention of treatment with etanercept is the change in TNF-α levels, as documented in a cohort of 41 non-systemic JIA patients with a median follow-up of 90 months (Kahn et al., 2016). Patients who experienced benefit and remained on treatment had more increase in their TNF-α levels at 6 weeks post-treatment onset than those who did not. TNF-α was detected in serum as complexes between etanercept (acting as decoy TNF-α receptor) and soluble TNF-α. This might not apply to other anti-TNF agents, as another study found that TNF-α and interleukin-17 (IL-17) levels during the first 6 months of treatment were significantly higher amongst JIA patients treated successfully with etanercept (n = 6) compared to adalimumab (n = 7) (Walters et al., 2016).

## Cell Biomarkers as Predictors of Response to Biologic Treatment

In RA, there is evidence of an increased percentage of regulatory T-cells (Treg) in responders to adalimumab compared to non-responders, therefore the Treg subpopulation has been suggested as a potential biomarker of response (McGovern et al., 2012; Nguyen et al., 2018)**.** A study in polyarticular JIA, including 30 patients treated with etanercept, methotrexate and prednisolone, explored the different Treg subsets in patients with active vs. inactive disease status and found that patients with active disease had a higher percentage of human leukocyte antigen-D related (HLA-DR) + Treg cells compared to patients with inactive disease (Rossetti et al., 2017). Interestingly, these Treg clonotypes were more closely related to synovial rather than circulating Treg cells and remained suppressive. Moreover, polyarticular and oligoarticular JIA patients on remission were found to have a significantly lower increase in the percentage of switched memory B-cells compared to active patients, during treatment with TNF inhibitors and methotrexate. On the other hand, patients on methotrexate alone had a similar rise in the frequency of this cell subset, irrespective of disease activity (Marasco et al., 2018), suggesting that switched memory B-cells could be a potential biomarker of response to biologic treatment in JIA.

## Genetic and Transcriptomic Biomarkers of Response to Biologic Treatment in JIA

Various genetic biomarkers have been investigated to assess their potential as predictor biomarkers of clinical response in JIA, with the majority of studies focused on response to methotrexate treatment as first line therapy in JIA (Hinks et al., 2011; Ramsey et al., 2019). Human leukocyte antigen B27 (HLA-B27) positivity in JIA patients was associated with double the odds of not being in clinical remission of treatment at the end of 8 years follow-up irrespective of treatment (Berntson et al., 2013). Despite previous studies identifying numerous single nucleotide polymorphisms (SNPs) at distinct loci associated with systemic JIA, only the high expression of IL1RN (the gene encoding IL1 receptor antagonist) alleles correlated strongly with lack of response to anakinra therapy (Arthur et al., 2018).

Analysis of gene expression profiles from SJIA achieving the adapted ACR JIA response criteria following initiation of treatment with canakinumab (including IL-1β, IL-1 receptors (IL1-R1 and IL1-R2), IL-1 receptor accessory protein (IL1-RAP), and IL-6) found the strongest clinical response was observed in patients with higher baseline expression of dysregulated genes and a strong early transcriptional response (Brachat et al., 2017). This suggests that successful treatment with canakinumab led to downregulation of innate immune response genes.

However, gene transcriptional profiling of peripheral blood mononuclear cells (PBMCs) of patients with polyarticular JIA with active disease vs. remission (on methotrexate monotherapy or methotrexate combined with biologic treatment) revealed underlying biologic differences which seem to represent a disease signature, as even JIA patients with well controlled disease had persistent transcriptomic differences compared to healthy children (Knowlton et al., 2009; Moncrieffe et al., 2010). The hepatocyte nuclear factor 4 alpha (HNF4α), which is expressed by T cells and granulocytes, emerged in another study as a key factor in controlling genes associated with JIA remission on treatment (including biologic therapies) (Jiang et al., 2013).

## Imaging Biomarkers of Response to Biologic Treatments

Imaging is most commonly used to confirm diagnosis, evaluate disease activity and response to therapy (Magni-Manzoni et al., 2009; Muller et al., 2009; Rebollo-Polo et al., 2011; Brown et al., 2012; Bugni Miotto e Silva et al., 2014). There is very little published on the use of imaging biomarkers to predict response and outcome of therapy. Ultrasound and magnetic resonance imaging (MRI) are the most commonly used imaging modalities/biomarkers to assess disease as they are sensitive to identifying inflammation and use non-ionizing radiation.

As far as ultrasound is concerned, one study with 42 JIA patients used a comprehensive (44-joint) power Doppler ultrasound (PDUS) assessment at 0, 3 and 6 months of starting additional DMARD or biologic treatment, in order to measure treatment response. A reduced 10-joint PDUS was deducted and found to have good sensitivity to change at 6 months of treatment (Collado et al., 2013). Another prospective study reported that the number of ultrasound positive joints (out of 28) decreased significantly after 24 weeks treatment with etanercept. The same study concluded that a higher number of ultrasound positive joints at baseline was seen in patients who achieved ACRPedi50 response compared to patients who did not, and that it was an independent predictive factor of response (odds ratio – OR = 1.438, 95% CI: 1.091–1.897) (Zhou and Gu, 2019). On the other hand, there are conflicting results as to whether positive ultrasound findings in JIA patients with inactive disease can predict flares, although it should be noted that a minority of patients were on biologic treatment in these studies (Magni-Manzoni et al., 2013; Miotto et al., 2017; De Lucia et al., 2018; Zhao et al., 2018).

Conventional MRI has been used in clinical trials to assess treatment response in RA (Woodworth et al., 2017), psoriatic arthritis (NCT03783026) and axial spondyloarthritis (van der Heijde et al., 2018). More specifically, the Outcome Measures in Rheumatology Clinical Trial (OMERACT) RA MRI score (RAMRIS), which evaluates the wrist and second to fifth metacarpophalangeal joints for osteitis, synovitis, erosions and joint space narrowing, is a valid biomarker in RA. It has demonstrated responsiveness, as early as 2 weeks post treatment (Conaghan et al., 2014) and is predictive of radiographic progression (Baker et al., 2014; Conaghan et al., 2014; Conaghan et al., 2019).

In a similar way, the Juvenile Arthritis Magnetic Resonance Imaging Score (JAMRIS) derives from MRI knee examination. Synovial hypertrophy, a component of the score, changed significantly in 15 consecutive JIA patients who were treated for 12 months with DMARDs and/or TNF-α blockers (Hemke et al., 2013). The Spondyloarthritis Research Consortium of Canada (SPARCC) scoring system for the assessment of sacroiliac joints has also been evaluated in juvenile spondyloarthritis; the standardized response mean calculated from paired MRI examinations before and after treatment (18/35 on biologic treatment) was moderate (Panwar et al., 2019). Moreover, a retrospective analysis of serial MRI scans of the sacroiliac joints in ERA patients, before and after initiation of TNF inhibitors, using again the SPARCC score, showed reduction of inflammation after treatment, but progression of structural damage (Bray et al., 2019). In addition to the aforementioned semi-quantitative scores, apparent diffusion coefficient (ADC) is a potential quantitative MRI biomarker for sacroiliitis (Vendhan et al., 2016). A study in patients with ERA treated with biologics showed that the reduction in ADC values after biologic treatment was greater in responders vs. non-responders (Bray et al., 2017).

There are inherent limitations in the usefulness of semi-quantitative scores as biomarkers of treatment response. The main drawback of ultrasound and MRI derived inflammation scores is that they are based on subjective interpretation of images by radiologists, which introduces bias and measurement error. This is more complicated when children are assessed, as the distinction between true inflammation and skeletal immaturity is challenging. On the other hand, quantitative imaging biomarkers are less operator-dependent and therefore have better reproducibility. Importantly, they offer a numerical value to facilitate comparison between serial scans. Although further work is needed for the technical and clinical validation of such biomarkers (Barendregt et al., 2019; Hall-Craggs et al., 2019; European Society of Radiology, 2020), they provide an opportunity for more robust measurement of treatment response and the ability to establish thresholds that guide clinical treatment.

## Various Predictor Biomarkers for Successful Withdrawal of Biologics Treatment in JIA

The ultimate goal for patients with JIA, as with other chronic diseases, is to achieve remission off medications, with obvious benefits for the patient as well as society, through improved productivity and reduced costs of health care. A systematic review of treatment withdrawal in JIA patients in remission described that the frequency of flares ranged from 30 to 100% in different studies (Halyabar et al., 2019). Data from a Canadian inception cohort showed that the probability of flare (defined as no longer fulfilling the criteria of inactive disease) within 12 months of attaining inactive disease was 42.5% and that of requiring treatment intensification was 26.6% (Guzman et al., 2016). After treatment withdrawal the corresponding numbers were 31.7 and 25%, although specifically for SJIA the risks were significantly lower, 6.2 and 3%, respectively. The identified risk factors for flares were RF positive polyarthritis, positive antinuclear antibodies (ANA) and features of severe disease before achieving inactive disease status, such as joint count over four or use of biologic treatment. In terms of long-term prognosis, results from the Nordic JIA study, an inception cohort study, suggested that only 32.8% (108/329) of participants achieved clinical remission (CR) defined as inactive disease without medications for 12 months, after 18 years of follow-up (Glerup et al., 2020). Patients with persistent oligoarticular and systemic-onset JIA achieved CR at the highest rate (54.2 and 53.8% respectively), in contrast to ERA, where only 8.1% of patients were successful. The systemic-onset category demonstrated also the highest probability of maintaining remission off biologic treatment, in comparison with other categories, in a multi-centre retrospective analysis (Simonini et al., 2018). In terms of the polyarticular JIA phenotype, a prospective study revealed that a significantly higher proportion of patients with RF positive polyarticular disease (7/17 or 40%) on anti-TNF therapy failed to maintain clinically inactive disease (CID) at 6 months, compared to patients with extended oligoarticular (1/18 or 6%) and RF negative polyarticular JIA (19/102 or 18%) (Lovell et al., 2018). Out of 107 patients who remained inactive for 6 months, 67 (63%) flared within 8 months of discontinuation of the biologic. Older age at disease onset, (hazard ratio–HR 0.92; 95%-CI 0.85–0.99), shorter disease duration (HR 1.12; 1.04–1.21), shorter duration from disease onset to achieving CID (HR 1.1; 95% CI 1.01–1.20) and shorter CID duration prior to discontinuation of biologic therapy (HR 1.16; 95%1.01, 1.33) were associated with a reduced likelihood of flaring. In a retrospective analysis which included only RF negative polyarticular and oligoarticular JIA types, positive ANA, male sex and raised CRP were identified as risk factors for flaring after discontinuation of etanercept, but could account only for 14% of the variability of the prediction (Aquilani et al., 2018). Shorter duration of etanercept treatment (6.1 vs. 15.8 months) before discontinuation and faster attainment of CID were recorded in patients who did not relapse after discontinuation of etanercept, compared to relapsers (Su et al., 2017). However, data from the Dutch Arthritis and Biologicals in Children (ABC) registry depicted the opposite association, which is that shorter duration of treatment (28.6 vs. 45 months) was recorded in the 15/39 patients who flared after stopping etanercept treatment as in remission (Otten et al., 2011a). In addition, data from a German biologic registry, showed that 11.7% of patients achieved drug-free remission at a mean follow-up of 9.1 years (Minden et al., 2019). In this study, they discovered that patients who initiated biologic treatment (etanercept in 91% cases) within 2 years of disease onset had higher chance of achieving remission off drugs (defined as clinical JADAS-10 score, assessing up to 10 active joints ≤1) at last follow-up, compared to others who started treatment between 2 and 5 years (OR: 0.28; 95% CI 0.12–0.64) or after 5 years (OR: 0.12; 95% CI 0.05–0.27) of disease onset. The researchers also demonstrated that earlier biologic treatment (<2 years) was associated with a higher proportion of patients with no functional limitations and optimal well-being in young adulthood compared to late treatment (>5 years). Furthermore, shorter disease duration (0.5 vs. 1.1 years) was associated with a successful gradual discontinuation of adalimumab in 29/35 patients with ERA, who had attained inactive disease and been on treatment for at least 2 years (Papailiou S et al., 2020). In contrast to the hopeful results of this retrospective study, data from the ABC registry revealed that despite the high rates of good response to etanercept in psoriatic JIA patients, 5/6 patients who ceased treatment at 22 months flared at a median of 2 months (Otten et al., 2011b). Finally, longer retention of the first biologic, as well as increased frequency of treatment suspension due to remission was observed in patients aged less than 16 years at the initiation of biologic therapy, as per data from a Spanish biologic registry (Bethencourt Baute et al., 2018).

As far as laboratory markers are concerned, the S100 proteins have been reported to not only predict response to biologic treatment, but also the risk of flaring post methotrexate and biologic treatment withdrawal (Foell et al., 2010; Anink et al., 2015). MRP8/14 above 720 ng/ml predicted flares within 6 months of discontinuation of etanercept in 26 patients with non-systemic JIA with an AUC of 0.75 (Anink et al., 2015). Moreover, higher levels of vascular endothelial growth factor (VEGF) and S100A12 were found in 9/22 of patients who relapsed after achieving remission, defined as absence of arthritic findings, disease activity score assessing 28 joints in RA (DAS-28) < 2.6, low CRP and matrix metalloproteinase-3 (MMP-3), on methotrexate and/or biologic treatment (Yamasaki et al., 2016). More specifically, S100A12 > 177 ng/ml and VEGF >158 pg/ml predicted relapse with 92.3 and 76.9% sensitivity, respectively and 77.8% specificity for both markers. Furthermore, raised levels of S100A12 during inactive disease was found to predict relapse with an AUC of 0.77 (Foell et al., 2004). On the other hand, two subsequent studies did not replicate these findings. In the first study, MRP8/14 or S100A12 were not significantly different between 39 patients with extended oligoarticular of polyarticular disease who flared within 8 months of anti-TNF treatment withdrawal and 67 patients who remained clinically inactive off biologic treatment (Hinze et al., 2019). In the other study, MRP8/14 was tested in two cohorts of non-systemic JIA patients, including 88 patients (27 on anti-TNF treatment) with inactive disease after 12 months of treatment (Barendregt et al., 2020). Levels of MRP8/14 did not predict the development of joint inflammation defined as an active joint count ≥1 at 6 or 12 months post treatment cessation on either cohort. A summary of evidence regarding MRP8/14 in non-systemic JIA created by the authors of the last study uncovered the discrepancies that exist between the published predictive models, which might be explained by the inconsistent definition of outcomes, dissimilar representation of JIA subtypes and treatments, as well as different assays used to measure serological biomarkers (Barendregt et al., 2020). With regards to systemic-onset JIA subtype, a study with 15 patients who stopped anakinra treatment after achieving an adapted ACRPedi90 response at 3 months, demonstrated that S100A12 levels were significantly raised in eight patients who relapsed later (Vastert et al., 2014). There is also limited evidence that higher MRP8/14 levels can predict relapse after anakinra withdrawal, based on two patients flaring and two who remaining inactive (Holzinger et al., 2012). Levels of the autoantibody targeting the oncoprotein DEK (anti-DEK) were found to be significantly elevated in 30 patients with polyarticular JIA who flared within 8 months after ceasing anti-TNF treatment, compared to 59 patients who did not flare. The difference in the anti-DEK levels between the groups was significant based on samples taken after patients flared, whilst anti-DEK levels at the time of discontinuation could not predict the outcome (Mor-Vaknin et al., 2018). Finally, an increased population frequency of an inflammatory CD4 memory subset (CD3^+^CD4^+^CD45RA^−^TNFα^+^) predicted relapse at 8 months after discontinuation of biologic therapy (AUC = 0.939) in polyarticular JIA patients with inactive disease prior to treatment cessation (Leong et al., 2019).

## Potential Clinical Use of Biologic Treatment-Related Biomarkers in SJIA

Biologic treatments have improved significantly the outcomes in SJIA. Controlling disease activity in SJIA is especially important, as active disease is associated with a higher risk for development of macrophage activation syndrome (MAS), which is a life-threatening complication. The treatment choices include the use of non-steroidal anti-inflammatory drugs (NSAIDs), glucocorticoids, methotrexate, anakinra, canakinumab, tocilizumab and TNFα blockers. There is no consensus on the treatment strategy, although anakinra is the biologic of choice when there are features of MAS (Ringold et al., 2013; Specialised Commissioning Team, 2015). Therefore, biomarkers predictive of response are needed to inform treatment decisions, in order to reduce the risk of complications related to the disease, but also diminish drug-related toxicity, particularly from steroids.

Several studies have reported clinical and laboratory findings that are associated with the achievement of inactive disease, the majority of them concerning therapy with anakinra (Table 2). Several biomarkers have been identified as useful in predicting the response to treatment with anakinra: early initiation of treatment increased the odds of achieving inactive disease and high neutrophil count at baseline was associated with good clinical response, whereas increased number of active joints at baseline was a negative prognostic factor for clinical improvement on treatment. Early treatment response appears to predict long-term response to both IL-1 and IL-6 blockers.

**TABLE 2:** Predictors for biologic treatment response in SJIA.

Ref	Medication	Study design	N patients	Results
Gattorno et al. (2008)	ANA	Prospective	22 (10 responders)	Complete responders had a lower number of active joints vs. non responders (median 3.5 vs. 7) and a higher number of neutrophils (median 19.3 vs. 9.1 × 10^3^/mm^3^),
Nigrovic et al. (2011)	ANA	Retrospective, multi-centre	46	Incomplete responders were younger at onset vs. complete responders (median age 5.2 vs. 10.2 years), (OR 1.5 per year; 95% CI 1.1–2.0)
Holzinger et al. (2012)	ANA, ETN	Prospective	52 12 on biologics	•MRP8/14 decreased markedly in responders to biologic treatment (12/12) and in responders (6/12) to methotrexate •MRP8/14 detects flares vs. inactive disease with outstanding diagnostic accuracy (AUC: 0.957 ± 0.019) •MRP8/14 > 740 ng/ml can predict relapse in next 6 months (AUC: 0.91), 13/26 inactive patients relapsed
Vastert et al. (2014)	ANA	Prospective single-centre	20 (15 responders)	•S100A8/9 (MRP8/14), S100A12 and IL-8 decreased markedly in responders (ACR Pedi 90) at 3 months •Lower levels of IL-8, S100A12, S100A8/9 at 3 months in 7/15 patients with ID who succeeded to discontinue treatment within a year (significant only for S100A12)
Pardeo et al. (2015)	ANA	Retrospective, single-centre	25 (14 responders)	Earlier treatment from disease onset associated with ID at 6 months (median 1.9 vs. 24.5 months).
Saccomanno et. al. (2019)	ANA	Retrospective single-centre	62 (24 responders)	Predictors of complete clinical response at 1 year included: •Disease duration ≤3.9 years (OR 6.78; 95% CI 1.30–35.27), •Active joint count ≤10 (OR 8.25; 95% CI 1.26–53.91), •Ferritin >444 ng/ml (OR 4.75; 95% CI 1.16–19.50), •Systemic manifestation score >3 (OR 6.44; 95% CI 1.38–24.62), AUC: 0.83
Kearsley-Fleet et al. (2019)	ANA, TCZ	Prospective, multi-centre	76	Baseline characteristics not associated with response (ACR Pedi 90, MDA or ID)
Ter Haar et al. (2019)	ANA	Prospective, single-centre	42 (32 ID at 1 year)	•ID at 1 month after ANA treatment predicted ID at 1 year (OR 27; 95% CI 4.17–539.74), AUC: 0.84 •Neutrophils>9 × 10^9^/L at baseline predict ID at 1 year (OR 38.67; 95% CI 6.53–362.73), AUC: 0.85
Ruperto et al. (2018)	CAN	Open-label, long-term extension study	144 (96 early responders)	Early responders (completed glucocorticoid tapering in part I of trial 2) achieved greater decrease in JADAS during the study as compared with late responders (mixed model; *p* < 0.01)
Bielak et al. (2018)	TCZ	Prospective, multi-centre	46	•7/17 (41%) patients showing inactive disease at the last visit had a response to TCZ within 5 weeks •Polycyclic course was associated with greater odds of clinical response (OR 7.0; 95% CI 1.8–27.2) compared to monocyclic or polyarticular course of SJIA

ANA, Anakinra; ACR, American college of Rheumatology; AUC, area under the curve; CAN, canakinumab; CI, confidence interval; ETN, etanercept; ID, clinically inactive disease; MDA, minimal disease activity; MRP, myeloid-related protein; OR, odds ratio; Pedi, pediatric; SJIA, systemic juvenile idiopathic arthritis; TCZ, tocilizumab.

The proinflammatory proteins MRP8/14 and S100A12 can be useful as diagnostic and therapeutic prognostic: both markers rise in active disease. The diagnostic accuracy of MRP8/14 exceeded the accuracy of established inflammatory markers such as CRP and ESR (Holzinger et al., 2012), whereas S100A12 can help differentiate between SJIA and other causes of systemic inflammation (Wittkowski et al., 2008). Moreover, their values decreased sharply in patients who displayed significant clinical improvement with treatment, such as fulfilling the ACRPedi90 criteria of response or the Wallace criteria of inactive disease (Holzinger et al., 2012; Vastert et al., 2014). Importantly, low levels during inactive disease were associated with successful tapering of anakinra, whilst levels of MRP8/14 above a cut-off were predictive of relapse (based on limited number of patients) (Holzinger et al., 2012; Vastert et al., 2014).

## Discussion

There have been previous reviews exploring the broad subject of biomarker identification in JIA (Consolaro et al., 2015; Gohar et al., 2016; Shoop-Worrall et al., 2019). This review exposed a diverse group of potential biomarkers, including inherent patient characteristics, clinical, laboratory, genetic, transcriptomic and imaging features, which are associated with short-term and long-term therapeutic goals, such as the attainment of inactive disease on biologic treatment and the sustainment of clinical remission after treatment withdrawal.

The JIA clinical phenotype, as defined by the ILAR classification is an important prognostic factor for long-term disease outcome as patients with persistent oligoarticular and systemic JIA subtypes are more likely to achieve remission without medications. However, JIA phenotype also influenced the response to biologic treatment as patients with persistent oligoarticular JIA had a higher chance to respond to etanercept than patients with polyarticular subtypes, whereas RF positive polyarticular category was associated with a higher risk of flares on anti-TNF treatment. As discussed above, longer disease duration at the onset of biologic treatment, higher CHAQ scores, concurrent steroid administration and previous use of multiple DMARDs are negative predictive factors of response to anti-TNF agents, suggesting that the timing of initiation of biologic treatment is crucial. Biologic treatment initiation early in the disease course was associated not only with better clinical response to etanercept and anakinra (the latter for patients with SJIA), but also with a higher chance of treatment discontinuation due to remission and better functional outcomes in young adulthood. This is an important observation as this is a factor that can be influenced by clinicians, whereas the same does not apply for the age of disease onset and JIA subtype. Moreover, clinical improvement within weeks from biologic initiation in patients with SJIA, but also in polyarticular JIA patients on adalimumab, is predictive of a future well-controlled disease.

All things considered, it should be noted that there is limited information about clinical predictors of response to biologics other than etanercept and anakinra, as there is longer experience with these biologic treatments in JIA, which is reflected in the available information from national JIA registries. This is also the reason for focusing our review on detailing biomarkers of response to etanercept across various JIA phenotypes and to anakinra in SJIA.

Nonetheless, there is no doubt that data from national registries have deepened our understanding about long-term outcomes of patients with JIA and have allowed us to assess the efficacy and safety of various biologic treatments and discover predictors of treatment success. There is immense potential from the development of national registries. Their growth will ensure that more extensive data can be collected, as efficiently as possible, while also expanding the collaboration and data sharing between nations as treatment recommendations and access to biologic treatment differ world-wide. One of the major challenges is ensuring that data collection is continued without interruptions during transition of JIA patients to adult care. The COVID-19 Global Rheumatology Alliance is a recent example of successful international collaboration resulting in the accumulation of important knowledge related to the risk of COVID-19 infection in immunosuppressed patients, which has informed the management of rheumatology patients during the pandemic (Robinson and Yazdany, 2020).

In terms of laboratory tests, MRP8/14 and S100A12 have emerged as the most promising biomarkers for predicting treatment response to methotrexate and bDMARDs, as well as indicating whether there is an increased risk of flare during inactive disease, which might deter clinicians from tapering treatment. However, not all studies have confirmed their ability to predict flares for non-systemic JIA patients and there was a small number of SJIA patients included. Moreover, the added value of MRP8/14 to the prediction model for treatment response based on clinical features alone was small; *R* ^2^ increased from 0.50 to 0.54 (Anink et al., 2015), raising further questions about their clinical utility. Further prospective studies with larger number of patients are needed to ratify these encouraging results. The findings from the interventional study PREDICT-JIA, which used S100A12 and high sensitivity CRP for treatment withdrawal stratification are expected in the near future (ISRCTN69963079).

More studies are also required to delineate the pharmacokinetics of biologics and examine whether the proactive measurement of trough levels, along with dose titration can improve patient outcomes and drug retention or support a safer tapering strategy. The presence of neutralizing ADA appears to be linked with potential loss of efficacy or infusion reactions, in the cases of adalimumab, infliximab and tocilizumab. However, many questions remain unanswered, such as if proactive monitoring of ADA and trough levels can reduce the risk of loss of response due to dose titration, or if in the light of secondary inefficacy, drug level and immunogenicity to biologic agents can aid the choice of the subsequent biologic treatment.

As far as imaging is concerned, there is paucity of validated imaging biomarkers in JIA, compared to RA. This might be explained by the different distribution of joint inflammation in JIA, often involving multiple large joints, which are more difficult to image, compared to the small joints of hands or feet alone often affected in RA. In addition, it is less feasible to organize scans for younger children with JIA. An imaging technique which offers whole-body coverage could be a logical option for assessment of JIA patients with different clinical presentation for the detection of subclinical synovitis. Whole-body MRI (WBMRI) with contrast has been used to assess for musculoskeletal inflammation in studies for RA, PsA and ankylosing spondylitis (AS) (Axelsen et al., 2014; Poggenborg et al., 2015), therefore we propose that this imaging technique could potentially have wide imaging biomarker utility across all JIA phenotypes. The value of MR imaging has been better appreciated in ERA. The presence of sacroiliitis on MRI is not only diagnostic, but helps to shape therapeutic decisions, as axial inflammation responds better to bDMARDs than conventional therapy. Moreover, improvement of sacroiliitis with treatment can be detected by MRI, suggesting that MRI is a sensitive to change imaging biomarker for response to biologic treatment. More recently, the use of quantitative imaging MRI techniques to objectify change in inflammation offers additional benefits (Hall-Craggs et al., 2018). More specifically, these measures are objective and reproducible as they are less dependent on the radiologist experience.

Ultrasound examination of multiple (eight) large joints using Power Doppler (PD) was feasible for assessment of patients with JIA, taking on average 30 min to complete (Zhao et al., 2018). Ultrasound should be able to provide a clear distinction between chronic synovitis defined as hypoechoic synovial hypertrophy commonly found in patients with longstanding disease and joint damage, and active synovitis, diagnosed by the presence of PD signal within the joint. However, in the above studies (Magni-Manzoni et al., 2013; De Lucia et al., 2018; Zhao et al., 2018), researchers compared clinical findings in JIA with positive ultrasound findings, which included gray scale and/or PD signal abnormalities. Interestingly, Magni-Manzoni et al. reported that PD signal was seen more frequently in the patients who stayed inactive than in patients who flared during follow-up (Magni-Manzoni et al., 2013). In comparison with adults, physiological intra-articular vascularity is a common finding in young people who are still to complete their growth, which makes the interpretation of joint inflammation in the context of JIA more challenging. In order to minimize over-reporting of active synovitis, the ultrasound OMERACT initiative amended the definitions of ultrasound-detected joint pathology in children (Collado et al., 2018). Although there is some evidence that ultrasound can detect reduction in joint inflammation after treatment, further research is needed to validate ultrasound as a tool to guide clinical management in patients with clinically inactive disease.

**FIGURE 1:**
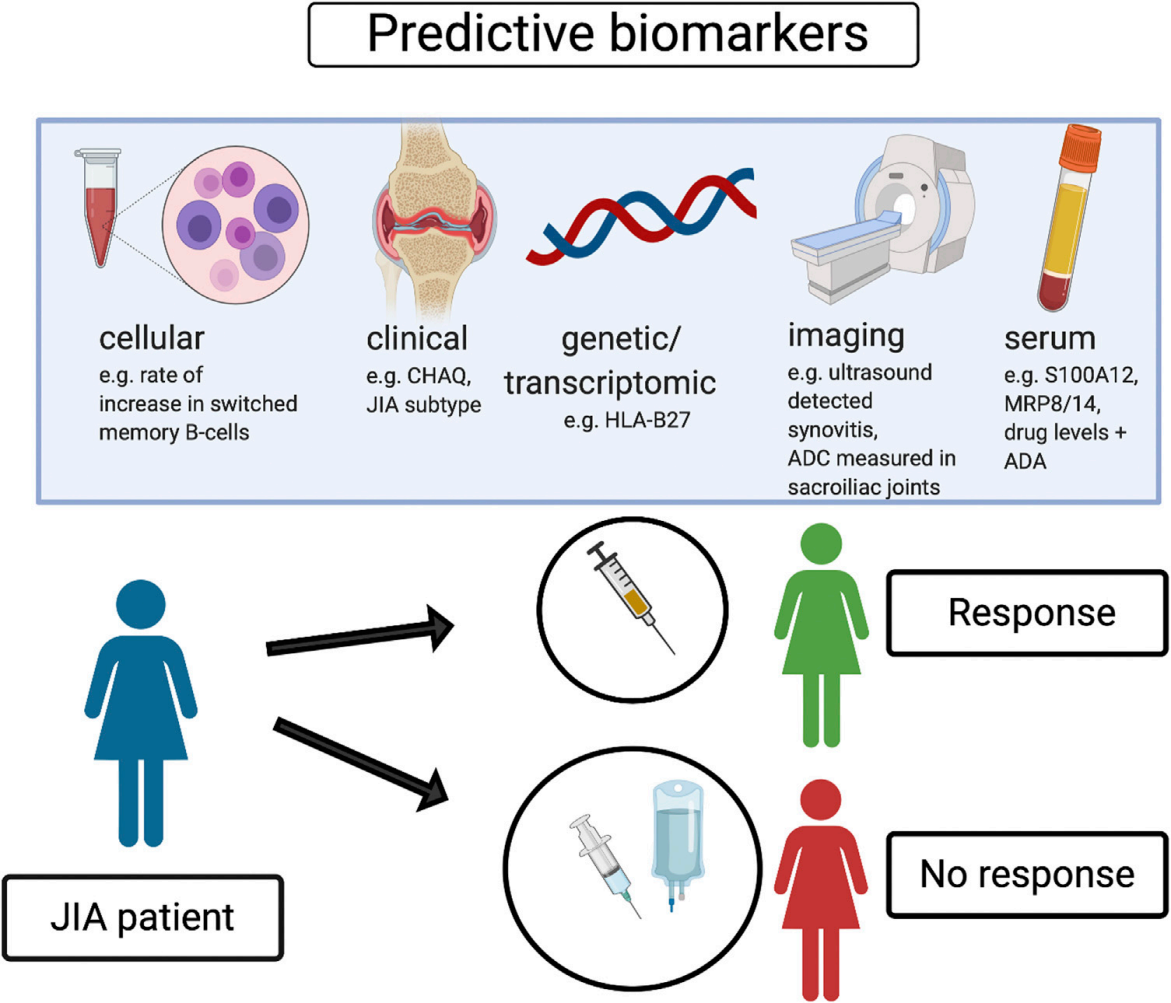
Potential predictive biomarkers of response to biologic treatment.

In conclusion, specific clinical features, serum proinflammatory proteins, selected cellular subsets and newly emerging transcriptomic signatures, in addition to imaging outcomes have been identified as potential positive or negative prediction markers of response to biologic treatment, as well as achievement of remission without treatment. Further research studies are needed to develop and validate individual or composite biomarkers with clinical applicability that could improve biologic treatment management in patients with JIA, as well as personalized treatment strategies. We propose some potential predictive biomarkers related to biologic treatment response in JIA which could be associated with patient benefit and optimization of treatment strategies (Figure 1).

## Author Contributions

Conception and design CC and VC. Literature review VC, BJ, MH-C, and CC. Drafting manuscript VC. Revising manuscript MH-C, BJ, CF, ML, LW, and CC. All authors approved the final version of the manuscript.

## Funding

This work is funded by an Action Medical grant to MHC and CC (1063), a Center of Excellence (Center for Adolescent Rheumatology vs. Arthritis) grant to LW and CC (21593) as well as grants from NIHR Biomedical Research Center at University College London Hospital to CC (BRC/III 525, BRC/III 773). LW and BJ are supported by grants from vs. Arthritis (20164 and 21593), MRC (MR/R013926/1), GOSH Children’s Charity and the NIHR Biomedical Research Center at GOSH. VC is supported by a grant from British Society of Rheumatology (4190033). CF is supported by a grant from vs. Arthritis (20762) and NIHR Biomedical Research Center at University College London Hospital. The views expressed are those of the authors and not necessarily those of the NHS, the NIHR or the Department of Health.

## Conflict of Interest

The authors declare that the research was conducted in the absence of any commercial or financial relationships that could be construed as a potential conflict of interest.

## References

[B1] Alberdi-Saugstrup M. Nielsen S. Mathiessen P. Nielsen C. H. Müller K. (2017). Low pretreatment levels of myeloid-related protein-8/14 and C-reactive protein predict poor adherence to treatment with tumor necrosis factor inhibitors in juvenile idiopathic arthritis. Clin. Rheumatol. 36, 67–75. 10.1007/s10067-016-3375-x 27562034

[B2] Alexeeva E. I. Namazova-Baranova L. S. Bzarova T. M. Valieva S. I. Denisova R. V. Sleptsova T. V. (2017). Predictors of the response to etanercept in patients with juvenile idiopathic arthritis without systemic manifestations within 12 months: results of an open-label, prospective study conducted at the National Scientific and Practical Center of Children’s Health, Russia. Pediatr. Rheumatol. Online J. 15, 51 10.1186/s12969-017-0178-9 28615036 PMC5471744

[B3] Anink J. Van Suijlekom-Smit L. W. A. Otten M. H. Prince F. H. M. Van Rossum M. A. J. Dolman K. M. (2015). MRP8/14 serum levels as a predictor of response to starting and stopping anti-TNF treatment in juvenile idiopathic arthritis. Arthritis Res. Ther. 17, 200 10.1186/s13075-015-0723-1 26249667 PMC4528380

[B4] Aquilani A. Marafon D. P. Marasco E. Nicolai R. Messia V. Perfetti F. (2018). Predictors of flare following etanercept withdrawal in patients with rheumatoid factor-negative juvenile idiopathic arthritis who reached remission while taking medication. J. Rheumatol. 45, 956–961. 10.3899/jrheum.170794 29717035

[B5] Arthur V. L. Shuldiner E. Remmers E. F. Hinks A. Grom A. A. Foell D. (2018). IL1RN variation influences both disease susceptibility and response to recombinant human interleukin-1 receptor antagonist therapy in systemic juvenile idiopathic arthritis. Arthritis Rheumatol. 70, 1319–1330. 10.1002/art.40498 29609200 PMC6105455

[B6] Axelsen M. B. Eshed I. Duer-Jensen A. Møller J. M. Pedersen S. J. Østergaard M. (2014). Whole-body MRI assessment of disease activity and structural damage in rheumatoid arthritis: first step towards an MRI joint count. Rheumatology (Oxford) 53, 845–853. 10.1093/rheumatology/ket425 24390938

[B7] Baker J. F. Ostergaard M. Emery P. Hsia E. C. Lu J. Baker D. G. (2014). Early MRI measures independently predict 1-year and 2-year radiographic progression in rheumatoid arthritis: secondary analysis from a large clinical trial. Ann. Rheum. Dis. 73, 1968–1974. 10.1136/annrheumdis-2013-203444 23904470

[B8] Barendregt A. M. Bray T. J. P. Hall-Craggs M. A. Maas M. (2019). Emerging quantitative MR imaging biomarkers in inflammatory arthritides. Eur. J. Radiol. 121, 108707 10.1016/j.ejrad.2019.108707 31707169

[B9] Barendregt A. M. Veldkamp S. R. Hissink Muller P. C. E. van de Geer A. Aarts C. van Gulik E. C. (2020). MRP8/14 and neutrophil elastase for predicting treatment response and occurrence of flare in patients with juvenile idiopathic arthritis. Rheumatology (Oxford) 59, 2392–2401. 10.1093/rheumatology/kez590 31904851 PMC7449815

[B10] Berntson L. Nordal E. Aalto K. Peltoniemi S. Herlin T. Zak M. (2013). HLA-B27 predicts a more chronic disease course in an 8-year followup cohort of patients with juvenile idiopathic arthritis. J. Rheumatol. 40, 725–731. 10.3899/jrheum.121257 23547219

[B11] Bethencourt Baute J. J. Sanchez-Piedra C. Ruiz-Montesinos D. Medrano San Ildefonso M. Rodriguez-Lozano C. Perez-Pampin E. (2018). Persistence and adverse events of biological treatment in adult patients with juvenile idiopathic arthritis: results from BIOBADASER. Arthritis Res. Ther. 20, 227 10.1186/s13075-018-1728-3 30305158 PMC6235210

[B12] Bielak M. Husmann E. Weyandt N. Haas J.-P. Hügle B. Horneff G. (2018). IL-6 blockade in systemic juvenile idiopathic arthritis–achievement of inactive disease and remission (data from the German AID-registry). Pediatr. Rheumatol. Online J. 16, 22 10.1186/s12969-018-0236-y 29622022 PMC5887199

[B13] Brachat A. H. Grom A. A. Wulffraat N. Brunner H. I. Quartier P. Brik R. (2017). Early changes in gene expression and inflammatory proteins in systemic juvenile idiopathic arthritis patients on canakinumab therapy. Arthritis Res. Ther. 19, 13 10.1186/s13075-016-1212-x 28115015 PMC5260050

[B14] Bray T. J. P. Vendhan K. Ambrose N. Atkinson D. Punwani S. Fisher C. (2017). Diffusion-weighted imaging is a sensitive biomarker of response to biologic therapy in enthesitis-related arthritis. Rheumatology (Oxford) 56, 399–407. 10.1093/rheumatology/kew429 27994095 PMC5850817

[B15] Bray T. J. P. Lopes A. Fisher C. Ciurtin C. Sen D. Hall-Craggs M. A. (2019). Sacroiliac joint ankylosis in young spondyloarthritis patients receiving biologic therapy: observation of serial magnetic resonance imaging scans. Arthritis Rheumatol. 71, 594–598. 10.1002/art.40750 30295426 PMC6915840

[B16] Brown A. Hirsch R. Laor T. Hannon M. J. Levesque M. C. Starz T. (2012). Do patients with juvenile idiopathic arthritis in clinical remission have evidence of persistent inflammation on 3T magnetic resonance imaging? Arthritis Care Res. (Hoboken) 64, 1846–1854. 10.1002/acr.21774 22740386 PMC4398346

[B17] Brunelli J. B. Silva C. A. Pasoto S. G. Saa C. G. S. Kozu K. T. Goldenstein-Schainberg C. (2020). Anti-adalimumab antibodies kinetics: an early guide for juvenile idiopathic arthritis (JIA) switching. Clin. Rheumatol. 39, 515–521. 10.1007/s10067-019-04798-6 31707543

[B18] Brunner H. I. Lovell D. J. Finck B. K. Giannini E. H. (2002). Preliminary definition of disease flare in juvenile rheumatoid arthritis. J. Rheumatol. 29, 1058–1064. 12022323

[B19] Brunner H. I. Ruperto N. Zuber Z. Keane C. Harari O. Kenwright A. (2015). Efficacy and safety of tocilizumab in patients with polyarticular-course juvenile idiopathic arthritis: results from a phase 3, randomised, double-blind withdrawal trial. Ann. Rheum. Dis. 74, 1110–1117. 10.1136/annrheumdis-2014-205351 24834925 PMC4431348

[B20] Bugni Miotto e Silva V. de Freitas Tavares da Silva C. de Aguiar Vilela Mitraud S. Nely Vilar Furtado R. Esteves Hilário M. O. Natour J. (2014). Do patients with juvenile idiopathic arthritis in remission exhibit active synovitis on joint ultrasound? Rheumatol. Int. 34, 937–945. 10.1007/s00296-013-2909-7 24318644

[B21] Collado P. Naredo E. Calvo C. Gamir M. L. Calvo I. García M. L. (2013). Reduced joint assessment vs. comprehensive assessment for ultrasound detection of synovitis in juvenile idiopathic arthritis. Rheumatology (Oxford) 52, 1477–1484. 10.1093/rheumatology/ket148 23620551

[B22] Collado P. Windschall D. Vojinovic J. Magni-Manzoni S. Balint P. Bruyn G. A. W. (2018). Amendment of the OMERACT ultrasound definitions of joints’ features in healthy children when using the Doppler technique. Pediatr. Rheumatol. Online J. 16, 23 10.1186/s12969-018-0240-2 29631610 PMC5892017

[B23] Conaghan P. G. Østergaard M. Troum O. Bowes M. A. Guillard G. Wilkinson B. (2019). Very early MRI responses to therapy as a predictor of later radiographic progression in early rheumatoid arthritis. Arthritis Res. Ther. 21, 214 10.1186/s13075-019-2000-1 31639034 PMC6805378

[B24] Conaghan P. G. Peterfy C. Olech E. Kaine J. Ridley D. Dicarlo J. (2014). The effects of tocilizumab on osteitis, synovitis and erosion progression in rheumatoid arthritis: results from the ACT-RAY MRI substudy. Ann. Rheum. Dis. 73, 810–816. 10.1136/annrheumdis-2013-204762 24525910 PMC3995246

[B25] Consolaro A. Giancane G. Schiappapietra B. Davì S. Calandra S. Lanni S. (2016). Clinical outcome measures in juvenile idiopathic arthritis. Pediatr. Rheumatol. Online J. 14, 23 10.1186/s12969-016-0085-5 27089922 PMC4836071

[B26] Consolaro A. Varnier G. C. Martini A. Ravelli A. (2015). Advances in biomarkers for paediatric rheumatic diseases. Nat. Rev. Rheumatol. 11, 265–275. 10.1038/nrrheum.2014.208 25512012

[B27] Davies R. Gaynor D. Hyrich K. L. Pain C. E. (2017). Efficacy of biologic therapy across individual juvenile idiopathic arthritis subtypes: a systematic review. Semin. Arthritis Rheum. 46, 584–593. 10.1016/j.semarthrit.2016.10.008 27914689

[B28] De Lucia O. Ravagnani V. Pregnolato F. Hila A. Pontikaki I. Gattinara M. (2018). Baseline ultrasound examination as possible predictor of relapse in patients affected by juvenile idiopathic arthritis (JIA). Ann. Rheum. Dis. 77, 1426–1431. 10.1136/annrheumdis-2017-211696 29437586

[B29] Doeleman M. J. H. Van Maarseveen E. Swart J. F. (2019). Immunogenicity of biologic agents in juvenile idiopathic arthritis: a systematic review and meta-analysis. Rheumatology (Oxford) 58, 1839–1849. 10.1093/rheumatology/kez030 30809664 PMC6758589

[B30] European Society of Radiology (2020). ESR statement on the validation of imaging biomarkers. Insights Imaging 11, 76 10.1186/s13244-020-00872-9 32500316 PMC7272524

[B31] Favalli E. G. Pontikaki I. Becciolini A. Biggioggero M. Ughi N. Romano M. (2017). Real-life 10-year retention rate of first-line anti-TNF drugs for inflammatory arthritides in adult- and juvenile-onset populations: similarities and differences. Clin. Rheumatol. 36, 1747–1755. 10.1007/s10067-017-3712-8 28597133

[B32] Foell D. Wittkowski H. Hammerschmidt I. Wulffraat N. Schmeling H. Frosch M. (2004). Monitoring neutrophil activation in juvenile rheumatoid arthritis by S100A12 serum concentrations. Arthritis Rheum. 50, 1286–1295. 10.1002/art.20125 15077313

[B33] Foell D. Wulffraat N. Wedderburn L. R. Wittkowski H. Frosch M. Gerss J. (2010). Methotrexate withdrawal at 6 vs. 12 months in juvenile idiopathic arthritis in remission: a randomized clinical trial. J. Am. Med. Assoc. 303, 1266–1273. 10.1001/jama.2010.375 20371785

[B34] Gattorno M. Piccini A. Lasigliè D. Tassi S. Brisca G. Carta S. (2008). The pattern of response to anti-interleukin-1 treatment distinguishes two subsets of patients with systemic-onset juvenile idiopathic arthritis. Arthritis Rheum. 58, 1505–1515. 10.1002/art.23437 18438814

[B35] Geikowski T. Becker I. Horneff G. German BIKER Registry Collaborative Study Group (2014). Predictors of response to etanercept in polyarticular-course juvenile idiopathic arthritis. Rheumatology (Oxford) 53, 1245–1249. 10.1093/rheumatology/ket490 24599916

[B36] Giannini E. H. Ruperto N. Ravelli A. Lovell D. J. Felson D. T. Martini A. (1997). Preliminary definition of improvement in juvenile arthritis. Arthritis Rheum. 40, 1202–1209. 10.1002/1529-0131(199707)40:7<1202::AID-ART3>3.0.CO;2-R 9214419

[B37] Glerup M. Rypdal V. Arnstad E. D. Ekelund M. Peltoniemi S. Aalto K. (2020). Long-term outcomes in juvenile idiopathic arthritis: eighteen years of follow-up in the population-based nordic juvenile idiopathic arthritis cohort. Arthritis Care Res. 72, 507–516. 10.1002/acr.23853 30762291

[B38] Gohar F. Anink J. Moncrieffe H. Van Suijlekom-Smit L. W. A. Prince F. H. M. van Rossum M. A. J. (2018). S100A12 is associated with response to therapy in juvenile idiopathic arthritis. J. Rheumatol. 45, 547–554. 10.3899/jrheum.170438 29335345 PMC5880729

[B39] Gohar F. Kessel C. Lavric M. Holzinger D. Foell D. (2016). Review of biomarkers in systemic juvenile idiopathic arthritis: helpful tools or just playing tricks? Arthritis Res. Ther. 18, 163 10.1186/s13075-016-1069-z 27411444 PMC4944486

[B40] Guzman J. Oen K. Huber A. M. Watanabe Duffy K. Boire G. Shiff N. (2016). The risk and nature of flares in juvenile idiopathic arthritis: results from the ReACCh-Out cohort. Ann. Rheum. Dis. 75, 1092–1098. 10.1136/annrheumdis-2014-207164 25985972

[B41] Hall-Craggs M. A. Bray T. J. P. Ciurtin C. Bainbridge A. (2019). Quantitative magnetic resonance imaging has potential for assessment of spondyloarthritis: arguments for its study and use. J. Rheumatol. 46, 541–542. 10.3899/jrheum.181049 31043500

[B42] Hall-Craggs M. A. Bray T. P. J. Bainbridge A. P. (2018). Quantitative imaging of inflammatory disease: are we missing a trick? Ann. Rheum. Dis. 77, 1689–1691. 10.1136/annrheumdis-2018-213614 29909376

[B43] Halyabar O. Mehta J. Ringold S. Rumsey D. G. Horton D. B. (2019). Treatment withdrawal following remission in juvenile idiopathic arthritis: a systematic review of the literature. Paediatr. Drugs 21, 469–492. 10.1007/s40272-019-00362-6 31673960 PMC7301222

[B44] Heiligenhaus A. Foeldvari I. Edelsten C. Smith J. R. Saurenmann R. K. Bodaghi B. (2012). Proposed outcome measures for prospective clinical trials in juvenile idiopathic arthritis-associated uveitis: a consensus effort from the multinational interdisciplinary working group for uveitis in childhood. Arthritis Care Res. (Hoboken) 64, 1365–1372. 10.1002/acr.21674 22438273

[B45] Hemke R. van Rossum M. A. van Veenendaal M. Terra M. P. Deurloo E. E. de Jonge M. C. (2013). Reliability and responsiveness of the juvenile arthritis MRI scoring (JAMRIS) system for the knee. Eur. Radiol. 23, 1075–1083. 10.1007/s00330-012-2684-y 23085866

[B46] Hinks A. Moncrieffe H. Martin P. Ursu S. Lal S. Kassoumeri L. (2011). Association of the 5-aminoimidazole-4-carboxamide ribonucleotide transformylase gene with response to methotrexate in juvenile idiopathic arthritis. Ann. Rheum. Dis. 70, 1395–1400. 10.1136/ard.2010.146191 21515602 PMC3128324

[B47] Hinze C. H. Foell D. Johnson A. L. Spalding S. J. Gottlieb B. S. Morris P. W. (2019). Serum S100a8/A9 and S100A12 levels in children with polyarticular forms of juvenile idiopathic arthritis: relationship to maintenance of clinically inactive disease during anti-tumor necrosis factor therapy and occurrence of disease flare after discontinuation of therapy. Arthritis Rheumatol. 71, 451–459. 10.1002/art.40727 30225949 PMC6393224

[B48] Holzinger D. Frosch M. Kastrup A. Prince F. H. Otten M. H. Van Suijlekom-Smit L. W. A. (2012). The Toll-like receptor 4 agonist MRP8/14 protein complex is a sensitive indicator for disease activity and predicts relapses in systemic-onset juvenile idiopathic arthritis. Ann. Rheum. Dis. 71, 974–980. 10.1136/annrheumdis-2011-200598 22267331

[B49] Hunter D. J. Losina E. Guermazi A. Burstein D. Lassere M. N. Kraus V. (2010). A pathway and approach to biomarker validation and qualification for osteoarthritis clinical trials. Curr. Drug Targets 11, 536–545. 10.2174/138945010791011947 20199395 PMC3261486

[B50] Ilowite N. Porras O. Reiff A. Rudge S. Punaro M. Martin A. (2009). Anakinra in the treatment of polyarticular-course juvenile rheumatoid arthritis: safety and preliminary efficacy results of a randomized multicenter study. Clin. Rheumatol. 28, 129–137. 10.1007/s10067-008-0995-9 18766426

[B51] Jiang K. Frank M. Chen Y. Osban J. Jarvis J. N. (2013). Genomic characterization of remission in juvenile idiopathic arthritis. Arthritis Res. Ther. 15, R100 10.1186/ar4280 24000795 PMC4062846

[B52] Kahn R. Berthold E. Gullstrand B. Schmidt T. Kahn F. Geborek P. (2016). Circulating complexes between tumour necrosis factor-alpha and etanercept predict long-term efficacy of etanercept in juvenile idiopathic arthritis. Acta Paediatr. 105, 427–432. 10.1111/apa.13319 26707699 PMC5066673

[B53] Kearsley-Fleet L. Beresford M. W. Davies R. de Cock D. Baildam E. Foster H. E. (2019). Short-term outcomes in patients with systemic juvenile idiopathic arthritis treated with either tocilizumab or anakinra. Rheumatology (Oxford) 58, 94–102. 10.1093/rheumatology/key262 30137641 PMC6293481

[B54] Kearsley-Fleet L. Davies R. Lunt M. Southwood T. R. Hyrich K. L. (2016). Factors associated with improvement in disease activity following initiation of etanercept in children and young people with Juvenile Idiopathic Arthritis: results from the British Society for Paediatric and Adolescent Rheumatology Etanercept Cohort Study. Rheumatology (Oxford) 55, 840–847. 10.1093/rheumatology/kev434 26721878 PMC4830911

[B55] Kearsley-Fleet L. Heaf E. Davies R. Baildam E. Beresford M. W. Foster H. E. (2020). Frequency of biologic switching and the outcomes of switching in children and young people with juvenile idiopathic arthritis: a national cohort study. Lancet Rheumatol. 2, e217–e226. 10.1016/S2665-9913(20)30025-4 32280951 PMC7134528

[B56] Knowlton N. Jiang K. Frank M. B. Aggarwal A. Wallace C. Mckee R. (2009). The meaning of clinical remission in polyarticular juvenile idiopathic arthritis: gene expression profiling in peripheral blood mononuclear cells identifies distinct disease states. Arthritis Rheum. 60, 892–900. 10.1002/art.24298 19248118 PMC2758237

[B57] Leong J. Y. Chen P. Yeo J. G. Ally F. Chua C. Nur Hazirah S. (2019). Immunome perturbation is present in patients with juvenile idiopathic arthritis who are in remission and will relapse upon anti-TNFα withdrawal. Ann. Rheum. Dis. 78, 1712–1721. 10.1136/annrheumdis-2019-216059 31540934 PMC6900250

[B58] Lovell D. J. Brunner H. I. Reiff A. O. Jung L. Jarosova K. Němcová D. (2020). Long-term outcomes in patients with polyarticular juvenile idiopathic arthritis receiving adalimumab with or without methotrexate. RMD Open 6, e001208 10.1136/rmdopen-2020-001208 32665432 PMC7425194

[B59] Lovell D. J. Giannini E. H. Reiff A. Cawkwell G. D. Silverman E. D. Nocton J. J. (2000). Etanercept in children with polyarticular juvenile rheumatoid arthritis. Pediatric Rheumatology Collaborative Study Group. N. Engl. J. Med. 342, 763–769. 10.1056/NEJM200003163421103 10717011

[B60] Lovell D. J. Giannini E. H. Reiff A. O. Kimura Y. Li S. Hashkes P. J. (2013). Long-term safety and efficacy of rilonacept in patients with systemic juvenile idiopathic arthritis. Arthritis Rheum. 65, 2486–2496. 10.1002/art.38042 23754188

[B61] Lovell D. J. Johnson A. L. Huang B. Gottlieb B. S. Morris P. W. Kimura Y. (2018). Risk, timing, and predictors of disease flare after discontinuation of anti-tumor necrosis factor therapy in children with polyarticular forms of juvenile idiopathic arthritis with clinically inactive disease. Arthritis Rheumatol. 70, 1508–1518. 10.1002/art.40509 29604189 PMC6115300

[B62] Magni-Manzoni S. Epis O. Ravelli A. Klersy C. Veisconti C. Lanni S. (2009). Comparison of clinical versus ultrasound-determined synovitis in juvenile idiopathic arthritis. Arthritis Rheum. 61, 1497–1504. 10.1002/art.24823 19877100

[B63] Magni-Manzoni S. Scirè C. A. Ravelli A. Klersy C. Rossi S. Muratore V. (2013). Ultrasound-detected synovial abnormalities are frequent in clinically inactive juvenile idiopathic arthritis, but do not predict a flare of synovitis. Ann. Rheum. Dis. 72, 223–228. 10.1136/annrheumdis-2011-201264 22736098

[B64] Makay B. Gücenmez Ö. A. Ünsal E. (2016). Inactive disease in enthesitis-related arthritis: association of increased body mass index. J. Rheumatol. 43, 937–943. 10.3899/jrheum.151208 26980582

[B65] Marasco E. Aquilani A. Cascioli S. Moneta G. M. Caiello I. Farroni C. (2018). Switched memory B cells are increased in oligoarticular and polyarticular juvenile idiopathic arthritis and their change over time is related to response to tumor necrosis factor inhibitors. Arthritis Rheumatol. 70, 606–615. 10.1002/art.40410 29316374

[B66] Marino A. Real-Fernández F. Rovero P. Giani T. Pagnini I. Cimaz R. (2018). Anti-adalimumab antibodies in a cohort of patients with juvenile idiopathic arthritis: incidence and clinical correlations. Clin. Rheumatol. 37, 1407–1411. 10.1007/s10067-018-4057-7 29508177

[B67] McErlane F. Beresford M. W. Baildam E. M. Thomson W. Hyrich K. L. (2013). Recent developments in disease activity indices and outcome measures for juvenile idiopathic arthritis. Rheumatology (Oxford) 52, 1941–1951. 10.1093/rheumatology/ket150 23630368

[B68] McGovern J. L. Nguyen D. X. Notley C. A. Mauri C. Isenberg D. A. Ehrenstein M. R. (2012). Th17 cells are restrained by Treg cells via the inhibition of interleukin-6 in patients with rheumatoid arthritis responding to anti-tumor necrosis factor antibody therapy. Arthritis Rheum. 64, 3129–3138. 10.1002/art.34565 22674488

[B69] Minden K. Horneff G. Niewerth M. Seipelt E. Aringer M. Aries P. (2019). Time of disease-modifying antirheumatic drug start in juvenile idiopathic arthritis and the likelihood of a drug-free remission in young adulthood. Arthritis Care Res. (Hoboken) 71, 471–481. 10.1002/acr.23709 30044538

[B70] Miotto E Silva V. B. Mitraud S. A. V. Furtado R. N. V. Natour J. Len C. A. Terreri M. T. S. E. L. R. A. (2017). Patients with juvenile idiopathic arthritis in clinical remission with positive power Doppler signal in joint ultrasonography have an increased rate of clinical flare: a prospective study. Pediatr. Rheumatol. Online J. 15, 80 10.1186/s12969-017-0208-7 29132381 PMC5683235

[B71] Mo X. Chen X. Ieong C. Zhang S. Li H. Li J. (2020). Early prediction of clinical response to etanercept treatment in juvenile idiopathic arthritis using machine learning. Front. Pharmacol. 11, 1164 10.3389/fphar.2020.01164 32848772 PMC7411125

[B72] Moncrieffe H. Hinks A. Ursu S. Kassoumeri L. Etheridge A. Hubank M. (2010). Generation of novel pharmacogenomic candidates in response to methotrexate in juvenile idiopathic arthritis: correlation between gene expression and genotype. Pharmacogenet. Genomics. 20, 665–676. 10.1097/FPC.0b013e32833f2cd0 20827233 PMC2963015

[B73] Moncrieffe H. Ursu S. Holzinger D. Patrick F. Kassoumeri L. Wade A. (2013). A subgroup of juvenile idiopathic arthritis patients who respond well to methotrexate are identified by the serum biomarker MRP8/14 protein. Rheumatology (Oxford) 52, 1467–1476. 10.1093/rheumatology/ket152 23620557

[B74] Mor-Vaknin N. Rivas M. Legendre M. Mohan S. Yuanfan Y. Mau T. (2018). High levels of DEK autoantibodies in sera of patients with polyarticular juvenile idiopathic arthritis and with early disease flares following cessation of anti-tumor necrosis factor therapy. Arthritis Rheumatol. 70, 594–605. 10.1002/art.40404 29287303 PMC5876119

[B75] Mori M. Takei S. Imagawa T. Imanaka H. Nerome Y. Higuchi R. (2012). Safety and efficacy of long-term etanercept in the treatment of methotrexate-refractory polyarticular-course juvenile idiopathic arthritis in Japan. Mod. Rheumatol. 22, 720–726. 10.1007/s10165-011-0578-5 22212889

[B76] Mourão A. F. Santos M. J. Melo Gomes J. A. Martins F. M. Mendonça S. C. Oliveira Ramos F. (2016). Effectiveness and long-term retention of anti-tumour necrosis factor treatment in juvenile and adult patients with juvenile idiopathic arthritis: data from Reuma.pt. Rheumatology (Oxford) 55, 697–703. 10.1093/rheumatology/kev398 26672905

[B77] Müller L. Kellenberger C. J. Cannizzaro E. Ettlin D. Schraner T. Bolt I. B. (2009). Early diagnosis of temporomandibular joint involvement in juvenile idiopathic arthritis: a pilot study comparing clinical examination and ultrasound to magnetic resonance imaging. Rheumatology (Oxford) 48, 680–685. 10.1093/rheumatology/kep068 19386819 PMC2681286

[B78] Nguyen D. X. Cotton A. Attipoe L. Ciurtin C. Doré C. J. Ehrenstein M. R. (2018). Regulatory T cells as a biomarker for response to adalimumab in rheumatoid arthritis. J. Allergy Clin. Immunol. 142, 978–980. 10.1016/j.jaci.2018.04.026 29935955 PMC6127034

[B79] Nigrovic P. A. Mannion M. Prince F. H. M. Zeft A. Rabinovich C. E. van Rossum M. A. J. (2011). Anakinra as first-line disease-modifying therapy in systemic juvenile idiopathic arthritis: report of forty-six patients from an international multicenter series. Arthritis Rheum. 63, 545–555. 10.1002/art.30128 21280009

[B80] Nordal H. H. Brun J. G. Hordvik M. Eidsheim M. Jonsson R. Halse A. K. (2016). Calprotectin (S100A8/A9) and S100A12 are associated with measures of disease activity in a longitudinal study of patients with rheumatoid arthritis treated with infliximab. Scand. J. Rheumatol. 45, 274–281. 10.3109/03009742.2015.1107128 26767827

[B81] Otten M. H. Prince F. H. Armbrust W. Ten Cate R. Hoppenreijs E. P. Twilt M. (2011a). Factors associated with treatment response to etanercept in juvenile idiopathic arthritis. J. Am. Med. Assoc. 306, 2340–2347. 10.1001/jama.2011.1671 22056397

[B82] Otten M. H. Prince F. H. Ten Cate R. van Rossum M. A. Twilt M. Hoppenreijs E. P. (2011b). Tumour necrosis factor (TNF)-blocking agents in juvenile psoriatic arthritis: are they effective? Ann. Rheum. Dis. 70, 337–340. 10.1136/ard.2010.135731 21068101

[B83] Panwar J. Tse S. M. L. Lim L. Tolend M. A. Radhakrishnan S. Salman M. (2019). Spondyloarthritis research Consortium of Canada scoring system for sacroiliitis in juvenile spondyloarthritis/enthesitis-related arthritis: a reliability, validity, and responsiveness study. J. Rheumatol. 46, 636–644. 10.3899/jrheum.180222 30709956

[B84] Papailiou S D. F. Tsolia M. N. Maritsi D. N. 2020 Attainment of inactive disease following discontinuation of adalimumab monotherapy in patients with era. 26th European Paediatric Rheumatology Congress, Virtual. Pediatric Rheumatology 11 10.1186/s12969-020-00469-y 34145201

[B85] Papamichael K. Vogelzang E. H. Lambert J. Wolbink G. Cheifetz A. S. (2019). Therapeutic drug monitoring with biologic agents in immune mediated inflammatory diseases. Expert Rev. Clin. Immunol. 15, 837–848. 10.1080/1744666X.2019.1630273 31180729

[B86] Pardeo M. Pires Marafon D. Insalaco A. Bracaglia C. Nicolai R. Messia V. (2015). Anakinra in systemic juvenile idiopathic arthritis: a single-center experience. J. Rheumatol. 42, 1523–1527. 10.3899/jrheum.141567 26034148

[B87] Petty R. E. Southwood T. R. Baum J. Bhettay E. Glass D. N. Manners P. (1998). Revision of the proposed classification criteria for juvenile idiopathic arthritis: durban, 1997. J. Rheumatol. 25, 1991–1994. 9779856

[B88] Poggenborg R. P. Eshed I. Østergaard M. Sørensen I. J. Møller J. M. Madsen O. R. (2015). Enthesitis in patients with psoriatic arthritis, axial spondyloarthritis and healthy subjects assessed by ‘head-to-toe’ whole-body MRI and clinical examination. Ann. Rheum. Dis. 74, 823–829. 10.1136/annrheumdis-2013-204239 24389294

[B89] Ramsey L. B. Moncrieffe H. Smith C. N. Sudman M. Marion M. C. Langefeld C. D. (2019). Association of SLCO1B1 *14 allele with poor response to methotrexate in juvenile idiopathic arthritis patients. ACR Open Rheumatol. 1, 58–62. 10.1002/acr2.1008 31777781 PMC6858017

[B90] Rebollo-Polo M. Koujok K. Weisser C. Jurencak R. Bruns A. Roth J. (2011). Ultrasound findings on patients with juvenile idiopathic arthritis in clinical remission. Arthritis Care Res. (Hoboken) 63, 1013–1019. 10.1002/acr.20478 21485021

[B91] Ringold S. Weiss P. F. Beukelman T. DeWitt E. M. Ilowite N. T. Kimura Y. (2013). 2013 update of the 2011 American College of Rheumatology recommendations for the treatment of juvenile idiopathic arthritis: recommendations for the medical therapy of children with systemic juvenile idiopathic arthritis and tuberculosis screening among children receiving biologic medications. Arthritis Rheum. 65, 2499–2512. 10.1002/art.38092 24092554 PMC5408575

[B92] Robinson P. C. Yazdany J. (2020). The COVID-19 Global Rheumatology Alliance: collecting data in a pandemic. Nat. Rev. Rheumatol. 16, 293–294. 10.1038/s41584-020-0418-0 32242121 PMC7117553

[B93] Rossetti M. Spreafico R. Consolaro A. Leong J. Y. Chua C. Massa M. (2017). TCR repertoire sequencing identifies synovial Treg cell clonotypes in the bloodstream during active inflammation in human arthritis. Ann. Rheum. Dis. 76, 435–441. 10.1136/annrheumdis-2015-208992 27311837 PMC5284348

[B94] Ruperto N. Brunner H. I. Quartier P. Constantin T. Wulffraat N. M. Horneff G. (2018). Canakinumab in patients with systemic juvenile idiopathic arthritis and active systemic features: results from the 5-year long-term extension of the phase III pivotal trials. Ann. Rheum. Dis. 77, 1710–1719. 10.1136/annrheumdis-2018-213150 30269054 PMC6241618

[B95] Ruperto N. Lovell D. J. Cuttica R. Wilkinson N. Woo P. Espada G. (2007). A randomized, placebo-controlled trial of infliximab plus methotrexate for the treatment of polyarticular-course juvenile rheumatoid arthritis. Arthritis Rheum. 56, 3096–3106. 10.1002/art.22838 17763439

[B96] Ruperto N. Lovell D. J. Cuttica R. Woo P. Meiorin S. Wouters C. (2010). Long-term efficacy and safety of infliximab plus methotrexate for the treatment of polyarticular-course juvenile rheumatoid arthritis: findings from an open-label treatment extension. Ann. Rheum. Dis. 69, 718–722. 10.1136/ard.2009.100354 20237125 PMC2946101

[B97] Ruperto N. Ravelli A. Falcini F. Lepore L. de Sanctis R. Zulian F. (1998). Performance of the preliminary definition of improvement in juvenile chronic arthritis patients treated with methotrexate. Italian Pediatric Rheumatology Study Group. Ann. Rheum. Dis. 57, 38–41. 10.1136/ard.57.1.38 9536821 PMC1752459

[B98] Saccomanno B. Tibaldi J. Minoia F. Bagnasco F. Pistorio A. Guariento A. (2019). Predictors of effectiveness of anakinra in systemic juvenile idiopathic arthritis. J. Rheumatol. 46, 416–421. 10.3899/jrheum.180331 30647180

[B99] Shoop-Worrall S. J. W. Wu Q. Davies R. Hyrich K. L. Wedderburn L. R. (2019). Predicting disease outcomes in juvenile idiopathic arthritis: challenges, evidence, and new directions. Lancet Child Adolesc. Health 3, 725–733. 10.1016/S2352-4642(19)30188-9 31331873

[B100] Simonini G. Ferrara G. Pontikaki I. Scoccimarro E. Giani T. Taddio A. (2018). Flares after withdrawal of biologic therapies in juvenile idiopathic arthritis: clinical and laboratory correlates of remission duration. Arthritis Care Res. (Hoboken) 70, 1046–1051. 10.1002/acr.23435 28973842

[B101] Skrabl-Baumgartner A. Erwa W. Muntean W. Jahnel J. (2015). Anti-adalimumab antibodies in juvenile idiopathic arthritis: frequent association with loss of response. Scand. J. Rheumatol. 44, 359–362. 10.3109/03009742.2015.1022213 25974288

[B102] Skrabl-Baumgartner A. Seidel G. Langner-Wegscheider B. Schlagenhauf A. Jahnel J. (2019). Drug monitoring in long-term treatment with adalimumab for juvenile idiopathic arthritis-associated uveitis. Arch. Dis. Child. 104, 246–250. 10.1136/archdischild-2018-315060 30026253

[B103] Solari N. Palmisani E. Consolaro A. Pistorio A. Viola S. Buoncompagni A. (2013). Factors associated with achievement of inactive disease in children with juvenile idiopathic arthritis treated with etanercept. J. Rheumatol. 40, 192–200. 10.3899/jrheum.120842 23204218

[B104] Soyfoo M. S. Roth J. Vogl T. Pochet R. Decaux G. (2009). Phagocyte-specific S100A8/A9 protein levels during disease exacerbations and infections in systemic lupus erythematosus. J. Rheumatol. 36, 2190–2194. 10.3899/jrheum.081302 19755614

[B105] Specialised Commissioning Team, N. E. (2015). Appendix A: suggested treatment flow-chart for JIA. Available: https://www.england.nhs.uk/commissioning/wp-content/uploads/sites/12/2015/07/appx-a-jia-e03Pd.pdf (Accessed November 13, 2020). Online

[B106] Su Y. Yang Y.-H. Chiang B. L. (2017). Treatment response to etanercept in methotrexate refractory juvenile idiopathic arthritis: an analysis of predictors and long-term outcomes. Clin. Rheumatol. 36, 1997–2004. 10.1007/s10067-017-3682-x 28540607

[B107] Sun H. Van L. M. Floch D. Jiang X. Klein U. R. Abrams K. (2016). Pharmacokinetics and pharmacodynamics of canakinumab in patients with systemic juvenile idiopathic arthritis. J. Clin. Pharmacol. 56, 1516–1527. 10.1002/jcph.754 27119439

[B108] Ter Haar N. M. van Dijkhuizen E. H. P. Swart J. F. van Royen-Kerkhof A. El Idrissi A. Leek A. P. (2019). Treatment to target using recombinant interleukin-1 receptor antagonist as first-line monotherapy in new-onset systemic juvenile idiopathic arthritis: results from a five-year follow-up study. Arthritis Rheumatol. 71, 1163–1173. 10.1002/art.40865 30848528 PMC6617757

[B109] Ungar W. J. Costa V. Burnett H. F. Feldman B. M. Laxer R. M. (2013). The use of biologic response modifiers in polyarticular-course juvenile idiopathic arthritis: a systematic review. Semin. Arthritis Rheum. 42, 597–618. 10.1016/j.semarthrit.2012.10.006 23337074

[B110] van der Heijde D. Sieper J. Maksymowych W. P. Lambert R. G. Chen S. Hojnik M. (2018). Clinical and MRI remission in patients with nonradiographic axial spondyloarthritis who received long-term open-label adalimumab treatment: 3-year results of the ABILITY-1 trial. Arthritis Res. Ther. 20, 61 10.1186/s13075-018-1556-5 29587851 PMC5870399

[B111] Vastert S. J. de Jager W. Noordman B. J. Holzinger D. Kuis W. Prakken B. J. (2014). Effectiveness of first-line treatment with recombinant interleukin-1 receptor antagonist in steroid-naive patients with new-onset systemic juvenile idiopathic arthritis: results of a prospective cohort study. Arthritis Rheumatol. 66, 1034–1043. 10.1002/art.38296 24757154

[B112] Vendhan K. Bray T. J. P. Atkinson D. Punwani S. Fisher C. Sen D. (2016). A diffusion-based quantification technique for assessment of sacroiliitis in adolescents with enthesitis-related arthritis. Br. J. Radiol. 89, 20150775 10.1259/bjr.20150775 26642308 PMC4986497

[B113] Verstegen R. H. J. McMillan R. Feldman B. M. Ito S. Laxer R. M. (2020). Towards therapeutic drug monitoring of TNF inhibitors for children with juvenile idiopathic arthritis: a scoping review. Rheumatology (Oxford) 59, 386–397. 10.1093/rheumatology/kez285 31335941

[B114] Wallace C. A. Ruperto N. Giannini E. Childhood Arthritis and Rheumatology Research Alliance, Pediatric.Rheumatology International Trials Organization, Pediatric Rheumatology Collaborative Study Group (2004). Preliminary criteria for clinical remission for select categories of juvenile idiopathic arthritis. J. Rheumatol. 31, 2290–2294. 15517647

[B115] Walters H. M. Pan N. Lehman T. J. A. Adams A. Kalliolias G. D. Zhu Y. S. (2016). The impact of disease activity and tumour necrosis factor-α inhibitor therapy on cytokine levels in juvenile idiopathic arthritis. Clin. Exp. Immunol. 184, 308–317. 10.1111/cei.12782 26934060 PMC4872381

[B116] Wittkowski H. Frosch M. Wulffraat N. Goldbach-Mansky R. Kallinich T. Kuemmerle-Deschner J. (2008). S100A12 is a novel molecular marker differentiating systemic-onset juvenile idiopathic arthritis from other causes of fever of unknown origin. Arthritis Rheum. 58, 3924–3931. 10.1002/art.24137 19035478 PMC2680303

[B117] Woodworth T. G. Morgacheva O. Pimienta O. L. Troum O. M. Ranganath V. K. Furst D. E. (2017). Examining the validity of the rheumatoid arthritis magnetic resonance imaging score according to the OMERACT filter-a systematic literature review. Rheumatology (Oxford) 56, 1177–1188. 10.1093/rheumatology/kew445 28398508 PMC5850856

[B118] Yamasaki Y. Takei S. Imanaka H. Nerome Y. Kubota T. Nonaka Y. 2016). Prediction of long-term remission of oligo/polyarticular juvenile idiopathic arthritis with S100A12 and vascular endothelial growth factor. Mod. Rheumatol. 26, 551–556. 10.3109/14397595.2015.1109784 26474088

[B119] Yanai H. Lichtenstein L. Assa A. Mazor Y. Weiss B. Levine A. (2015). Levels of drug and antidrug antibodies are associated with outcome of interventions after loss of response to infliximab or adalimumab. Clin. Gastroenterol. Hepatol. 13, 522–530. 10.1016/j.cgh.2014.07.029 25066837

[B120] Yokota S. Imagawa T. Mori M. Miyamae T. Takei S. Iwata N. (2014). Longterm safety and effectiveness of the anti-interleukin 6 receptor monoclonal antibody tocilizumab in patients with systemic juvenile idiopathic arthritis in Japan. J. Rheumatol. 41, 759–767. 10.3899/jrheum.130690 24634205

[B121] Zanwar A. Phatak S. Aggarwal A. (2018). Prospective validation of the juvenile spondyloarthritis disease activity index in children with enthesitis-related arthritis. Rheumatology (Oxford) 57, 2167–2171. 10.1093/rheumatology/key246 30107576

[B122] Zhao Y. Rascoff N. E. Iyer R. S. Thapa M. Reichley L. Oron A. P. (2018). Flares of disease in children with clinically inactive juvenile idiopathic arthritis were not correlated with ultrasound findings. J. Rheumatol. 45, 851–857. 10.3899/jrheum.170681 29606669

[B123] Zhou L. Gu X. (2019). Correlation of ultrasonography synovitis with disease activity and clinical response to etanercept treatment in juvenile idiopathic arthritis patients. Braz. J. Med. Biol. Res. 52, e8565 10.1590/1414-431X20198565 31778437 PMC6886362

